# Dosing Regimen Optimization of Aztreonam/Avibactam According to Renal Function Stratification: A Population Pharmacokinetic-Guided Simulation Study

**DOI:** 10.3390/antibiotics15060576

**Published:** 2026-06-05

**Authors:** Ping Yang, Xianhua Zhang, Yufei Chen, Congya Zhou, Suodi Zhai

**Affiliations:** 1 Department of Pharmacy, Peking University Third Hospital, Beijing 100191, China; guoguokate@163.com (P.Y.); zhangxianhua432@163.com (X.Z.); chenyufeidx@163.com (Y.C.); zhoucongya@sina.com (C.Z.); 2 Therapeutic Drug Monitoring and Clinical Toxicology Center of Peking University, Beijing 100191, China; 3 Department of Nephrology, Longhua Hospital Affiliated to Shanghai University of Traditional Chinese Medicine, Shanghai 200032, China; 4 Institute for Drug Evaluation, Peking University Health Science Center, Beijing 100191, China

**Keywords:** aztreonam/avibactam, population pharmacokinetics, Monte Carlo simulation, probability of target attainment, renal impairment, dose optimization

## Abstract

**Background/Objectives**: Aztreonam/avibactam is a promising treatment option for serious infections caused by metallo-β-lactamase-producing carbapenem-resistant Enterobacterales (MBL-CRE). However, the labeled regimen is operationally demanding because it requires frequent, prolonged infusions, and the recommended loading dose does not match the commercially available vial strength. This population pharmacokinetic (PopPK)-based Monte Carlo simulation study aimed to optimize aztreonam/avibactam dosing across renal function strata while maintaining pharmacokinetic/pharmacodynamic (PK/PD) target attainment. **Methods**: Published PopPK models for aztreonam and avibactam were reconstructed and applied in Monte Carlo simulations. Virtual adult patients (body weight 70 kg) were stratified into five renal function groups according to creatinine clearance (CrCL): 101–120, 81–100, 51–80, 31–50, and 15–30 mL/min. Simulated scenarios varied infusion duration, dosing interval, maintenance dose, and loading strategy. Prespecified PK/PD targets were 60% fT > MIC (the percentage of dosing interval that free drug concentration remains above the minimum inhibitory concentration) for aztreonam (MIC 8 mg/L) and 50% fT > CT (the percentage of dosing interval that free drug concentration remains above the critical threshold concentration) for avibactam (CT 2.5 mg/L). A joint probability of target attainment (PTA) ≥ 90% was considered acceptable. **Results**: Regimen performance differed across renal function strata. For patients with CrCL > 80 mL/min, the labeled q6h regimen infused over 3 h remained the most robust option, whereas shortening the infusion to 1 h or 2 h reduced target attainment. In the CrCL 51–80 and 31–50 mL/min subgroups, both q6h/3 h and q6h/2 h regimens generally achieved acceptable PTA. However, in the CrCL 31–50 mL/min subgroup receiving q6h/2 h administration, omitting a loading dose was associated with reduced early avibactam exposure. In the CrCL 15–30 mL/min subgroup, a simplified half-vial regimen (0.75/0.25 g q8h/2 h) provided PTA comparable to that of the complex labeled reduced-dose regimen. Across loading dose scenarios, omission of the loading dose was best supported in the CrCL 51–80 mL/min subgroup, whereas retaining the labeled loading dose remained the more prudent approach in the CrCL 31–50 mL/min subgroup when a 2 h infusion was used. **Conclusions**: PopPK-guided, renal function-stratified simplification of aztreonam/avibactam dosing may improve clinical practicality without materially compromising PK/PD target attainment in selected patient subgroups. A 2 h infusion appears a reasonable alternative for patients with CrCL 31–80 mL/min, and a 0.75/0.25 g q8h/2 h half-vial regimen may be considered a plausible exploratory option for patients with CrCL 15–30 mL/min. These findings support more feasible administration strategies, but prospective clinical validation remains necessary.

## 1. Introduction

Carbapenem-resistant Enterobacterales (CRE) constitute a persistent and escalating therapeutic impasse in modern infectious disease practice. Notably, metallo-β-lactamase (MBL)-producing CRE exhibit broad-spectrum resistance to nearly all β-lactam agents, leaving few effective treatment options for severe infections [1,2]. The availability of the aztreonam/avibactam (ATM/AVI) fixed-dose combination marks a critical therapeutic advance, with avibactam protecting aztreonam from serine β-lactamase-mediated degradation and reconstituting its activity against MBL-positive strains co-expressing other β-lactamases [3,4]. Amid the relentless dissemination of MBL-mediated resistance worldwide, ATM/AVI has rapidly evolved into a first-line therapeutic cornerstone for multidrug-resistant Gram-negative infections.

Aztreonam/avibactam is a newly fixed-dose antimicrobial combination approved in China by the National Medical Products Administration on 24 June 2025 for intravenous use as aztreonam-avibactam sodium (Sufno^®^). Like other β-lactam-based regimens, ATM/AVI efficacy is driven by maintenance of exposure above predefined pharmacodynamic thresholds for an adequate proportion of the dosing interval [5,6]. The currently labeled regimen requires administration every 6 h with a 3 h infusion, and in patients with severe renal impairment, the dosing interval is prolonged, and a loading dose is recommended to accelerate target attainment [7]. Although pharmacologically sound, this approach is operationally complex in routine care. Repeated prolonged infusions increase nursing and pharmacy workload, constrain patient mobility, and disrupt inpatient care workflows. Furthermore, the recommended loading dose is misaligned with commercial vial presentations, predisposing to drug wastage, compounding inaccuracies, and inconsistent real-world implementation.

Despite its pivotal clinical role, the optimal ATM/AVI dosing strategy across the full spectrum of renal function remains incompletely defined. Contemporary PK/PD investigations have predominantly focused on patients with preserved renal function or limited renal subgroups, lacking systematic evaluation spanning the entire range of renal impairment (creatinine clearance: 15–120 mL/min) [8]. As a well-established framework for evaluating antimicrobial dosing strategies, population pharmacokinetic (PopPK) modeling combined with Monte Carlo simulation enables the quantification of target attainment probability (PTA) under different clinical scenarios. This approach facilitates systematic assessment of PTA across heterogeneous patient populations, accounting for interindividual pharmacokinetic variability, and furnishes data-driven evidence to guide individualized dosing decisions [9,10]. Critically, model-informed simulations can identify simplified regimens that preserve therapeutic efficacy while mitigating clinical complexity—an unmet clinical need for ATM/AVI. This approach not only ensures the rationality of dosing regimens but also facilitates the optimization of therapeutic outcomes for ATM/AVI combination therapy.

Herein, we reconstructed validated PopPK models of aztreonam and avibactam and performed Monte Carlo simulations to evaluate whether clinically pragmatic dosing simplification could be achieved without materially compromising PK/PD target attainment across renal function strata. The primary objective was to delineate simplified administration strategies that retain robust pharmacodynamic performance while enhancing clinical feasibility, reducing operational burden, and minimizing drug wastage in routine practice.

## 2. Results

### 2.1. Reconstruction of the Population Pharmacokinetic Models

Published two-compartment PopPK models for aztreonam and avibactam were successfully reconstructed. The model structure and associated covariate relationships were summarized in Table 1 [11]. In keeping with the source model, creatinine clearance (CrCL) was retained as the primary covariate on clearance. For CrCL > 80 mL/min, clearance increased linearly with CrCL; for CrCL ≤ 80 mL/min, the CrCL–clearance relationship was described by a power function.

### 2.2. External Validation of the Models

The external validation results are presented in Table 2. For aztreonam/avibactam combination therapy in infected patients, the reconstructed models yielded Mean Prediction Error percentage (MPE%) values of 16.8% and −18.8%, mean Absolute Prediction Error percentage (MAPE%) values of 18.4% and 16.6%; proportions of predicted concentrations within 20% of observed values (F_20_)of 50% and 66.7%, and proportions of predicted concentrations within 30% of observed values (F_30_) of 100% and 66.7%, respectively. For aztreonam monotherapy in healthy volunteers, the model demonstrated MPE% of 13.9%, MAPE% of 21.8%, F_20_ of 63.6%, and F_30_ of 100%. These findings indicate that the reconstructed popPK models provided accurate predictions of plasma concentrations that were in good agreement with observed values across different scenarios, including both combination therapy and aztreonam monotherapy, as well as single-dose and multi-dose administration schedules. The validation populations encompassed both healthy subjects and infected patients. Due to the limited availability of published concentration-time data, only accessible datasets were included in the external validation. The results met standard criteria for external validation of population pharmacokinetic models, confirming satisfactory predictive performance and generalizability of the model. These findings provide a solid foundation for the subsequent Monte Carlo simulation analyses.

### 2.3. Regimen Performance Across Renal Function Strata

**Figure 1:**
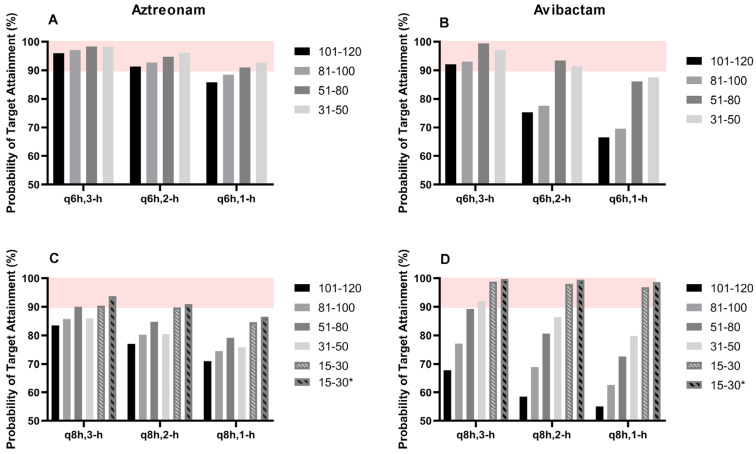
Probability of target attainment (PTA) for aztreonam (ATM) and avibactam (AVI) under different dosing regimens across renal function subgroups. (**A**) ATM q6h regimens; (**B**) AVI q6h regimens; (**C**) ATM q8h regimens; (**D**) AVI q8h regimens. Bars are coded by grayscale and fill pattern to distinguish creatinine clearance (CrCL) groups (mL/min): 101–120, 81–100, 51–80, 31–50, 15–30, and 15–30*. Maintenance doses were administered according to the labeled dose in the package insert, except for the CrCL 15–30* mL/min subgroup, which received a half-vial adjusted maintenance dose. The pink shaded area indicates the region where PTA meets the prespecified acceptable criterion (≥90%). Abbreviations: ATM, aztreonam; AVI, avibactam; CrCL, creatinine clearance; q6h, every 6 h; q8h, every 8 h; h, hour; PTA, probability of target attainment.

Monte Carlo simulations showed clear renal function-dependent differences in regimen performance. The PTA for aztreonam/avibactam regimens under different scenarios was shown in Figure 1. For patients with CrCL > 80 mL/min, the labeled maintenance dose regimen (1.5/0.5 g q6h infused over 3 h) remained the most robust option. Shortening the infusion to 1 h or 2 h reduced PTA, particularly for avibactam. In the CrCL 31–50 and 51–80 mL/min subgroups, both the q6h/3 h and q6h/2 h regimens maintained acceptable PTA and therefore appeared hypothetically suitable from a PK/PD perspective. In the CrCL 15–30 mL/min subgroup, the simplified 0.75/0.25 g q8h/2 h regimen provided PTA comparable to that of the complex labeled reduced-dose regimen (0.675/0.225 g q8h/3 h), suggesting that it may be considered a packaging-matched alternative.

### 2.4. Loading Dose Evaluation

Loading dose effects were evaluated in the CrCL 31–100 mL/min subgroups receiving q6h maintenance dosing. The detailed results are summarized in Table 3. Overall, concentration-time profiles converged rapidly after maintenance dosing, indicating that the influence of the loading strategy was mainly confined to early exposure. In the CrCL 51–80 mL/min subgroup, omission of the loading dose had little effect on PTA, including under the q6h/2 h regimen.

Notably, under the simulation of some dosing regimens (including the labeled recommended dose regimen), the joint PTA of avibactam at the second dose ranged between 89% and 90%; however, this did not affect the achievement of joint PTA above 90% at steady state after the 3rd to 4th doses. In contrast, in the subgroup with CrCL of 31–50 mL/min, when the q6h/2 h regimen was adopted without a loading dose, the avibactam PTA after both the first and second doses was below 90%. In comparison, the administration of either the labeled recommended loading dose or the packaging-matched loading dose of 1.5/0.5 g could more stably maintain the early drug exposure level. For patients with acute infections, rapid achievement of adequate drug exposure is crucial; therefore, the administration of a loading dose is recommended in such cases.

**Figure 2:**
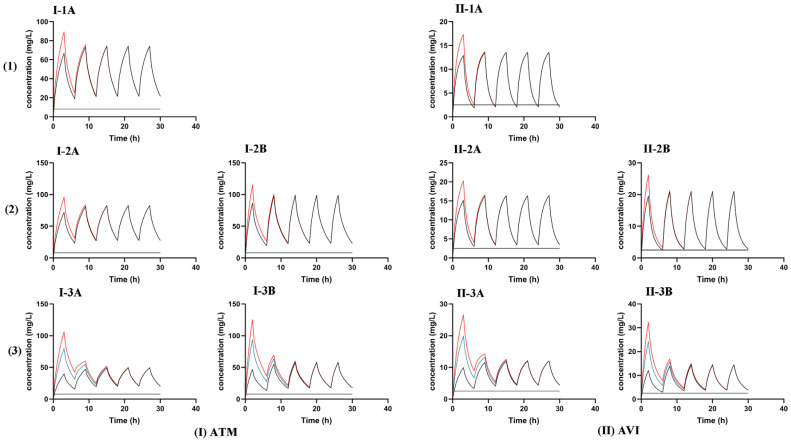
Mean concentration–time profiles of aztreonam ((**I**) ATM) and avibactam ((**II**) AVI) under different loading dose strategies across renal function strata. Panels (**1**–**3**) represent creatinine clearance (CrCL) ranges of 81–100, 51–80, and 31–50 mL/min, respectively. Panels (**A**,**B**) denote q6h dosing with 3 h infusion and q6h dosing with 2 h infusion, respectively. The red line represents the 2.0/0.67 g loading dose (aztreonam/avibactam), the blue line represents the 1.5/0.5 g loading dose, and the black line represents no loading dose. Abbreviations: ATM, aztreonam; AVI, avibactam; CrCL, creatinine clearance; q6h, every 6 h; h, hour.

These findings suggest that loading simplification is not equally applicable across renal strata and should be considered selectively. Mean concentration-time curves under simulated scenarios with different loading doses are shown in Figure 2.

## 3. Discussion

This study used published PopPK models and Monte Carlo simulations to examine whether aztreonam/avibactam dosing can be simplified without materially compromising PK/PD target attainment. Formal sensitivity analysis was not conducted for the developed PopPK models. However, key parameters, including creatinine clearance stratification, minimum inhibitory concentration (MIC), and PK/PD targets, were selected in accordance with internationally recognized clinical standards. Furthermore, external validation of the model was performed and presented in the Section 2 and Section 4, using published plasma concentration data of aztreonam and avibactam following administration [12,13] as the external validation dataset. Statistical comparisons between observed and simulated concentrations yielded acceptable agreement, indicating that the model is generally robust with reliable predictive performance.

Virtual adult patients in our models were simulated with a fixed body weight of 70 kg, which represents the WHO standard reference weight for adult pharmacokinetic simulations. This fixed weight enabled direct and unbiased comparisons with previously published population pharmacokinetic studies of aztreonam/avibactam [11]. Given that the primary objective of this analysis was to evaluate the impact of renal function on drug exposure and target attainment, body weight was held constant to eliminate potential confounding effects and isolate the influence of renal clearance. Age was not included as an independent covariate in the current PopPK model, since the original validated model confirmed no significant effect of age on clearance after adjusting for CrCL and body weight.

Infection type was not incorporated as a covariate in the present analysis, mainly because the original PopPK models established in previous studies did not identify any statistically significant effect of infection type on drug clearance. Furthermore, the official prescribing information for aztreonam/avibactam does not recommend distinct dosing regimens or administration frequencies according to infection categories, with only treatment duration adjusted for different infectious scenarios.

Clinically, critically ill ICU patients frequently present with augmented renal clearance (ARC) or acute kidney injury (AKI). Meanwhile, sepsis, capillary leakage and fluid overload commonly lead to increased volume of distribution, which substantially alters in vivo drug disposition. Such pathophysiological changes may result in actual drug exposure and target attainment rates differing from simulation predictions. In this regard, the results of the current study should be interpreted as conditional estimates based on the modeled population, and extrapolation to ICU settings should be undertaken cautiously. Future modeling analyses may further introduce indicators reflecting infection severity and acute kidney injury status as key covariates and develop dedicated population pharmacokinetic models tailored specifically for ICU patients to further optimize dosing strategies.

The PK/PD targets selected in this study were based on well-established clinical and regulatory criteria. For aztreonam, 60% fT > MIC with an MIC of 8 mg/L represents a clinically validated breakpoint for aztreonam/avibactam against Enterobacterales, including MBL-producing CRE. For avibactam, 50% fT > CT at a threshold concentration of 2.5 mg/L is the established PK/PD threshold required to maintain sustained β-lactamase inhibition. This target has been validated in multiple prior PK/PD investigations [14,15] and was adopted in the registration trials for aztreonam/avibactam. Collectively, these targets correlate reliably with bacterial eradication and favorable clinical outcomes in real-world severe infections and are routinely applied in population pharmacokinetic simulations to optimize dosing for β-lactam/β-lactamase inhibitor combinations.

Three clinically relevant messages emerge.

First, model simulations suggest that a 3 h infusion may be preferable in patients with preserved renal function, whereas a 2 h infusion appears acceptable in selected patients with mild-to-moderate renal impairment. This pattern is biologically plausible for time-dependent β-lactam regimens [4]. In patients with higher renal clearance, shortening the infusion reduces the time during which free concentrations remain above the pharmacodynamic target, whereas reduced clearance in renal impairment partly offsets this effect. Our findings therefore provide preliminary support for renal function-specific administration rather than a uniform infusion duration across all patients, warranting additional clinical evaluation.

Second, the simplified half-vial regimen may offer potential practical advantages for patients with CrCL 15–30 mL/min. Although the labeled reduced-dose regimen is pharmacologically reasonable, it does not align with the available commercial presentation. If supported in prospective studies, the 0.75/0.25 g q8h/2 h regimen may reduce preparation complexity and drug waste while potentially preserving the expected PK/PD exposure profile.

Third, the need for a loading dose appears to depend on renal function and infusion strategy. In the CrCL 51–80 mL/min subgroup, omission of the loading dose had minimal effect on PTA, suggesting that simplification may be feasible in this subgroup. Fromage et al. also reported that reducing the loading dose of aztreonam/avibactam may be feasible without materially compromising PK/PD performance under selected conditions [10]. By contrast, in the CrCL 31–50 mL/min subgroup, early avibactam exposure under q6h/2 h remained sensitive to loading strategy, suggesting that retention of the labeled loading dose may be a more cautious approach in this population. These findings are consistent with the short half-lives of aztreonam and avibactam and the rapid convergence of exposure after repeated dosing.

This study contributes to the existing literature by focusing on clinically implementable simplified dosing regimens rather than solely on model development. In contrast to the present study, Fromage et al. [10] focused only on patients with normal renal function (CrCL > 80 mL/min), evaluating the feasibility of reducing the loading dose to minimize drug waste, with a fixed 3 h infusion. Their analysis mainly compared early and steady-state AUC. The current study extends these findings by covering the full spectrum of renal impairment (CrCL 15–120 mL/min) and systematically optimizing infusion duration, dosing interval, maintenance dose, and loading strategy.

The renal function-stratified framework could, with additional validation, be considered for future translation into bedside prescribing algorithms and integration into routine pharmacy workflows. From a clinical perspective, the labeled aztreonam/avibactam regimen, which requires administration every six hours with a three-hour prolonged infusion and individualized loading doses based on renal function, is logistically demanding in routine practice. The renal function-stratified simplified regimens proposed in this study include a 2 h infusion, a half-vial maintenance dose, and selective loading dose administration. These regimens facilitate clinical preparation, administration, and treatment adherence. Importantly, they maintain PK/PD target attainment with a PTA of at least 90%, while reducing nursing workload, minimizing drug waste, and lowering the risk of preparation errors, which directly improves clinical operability. Furthermore, the findings provide a critical PK/PD foundation for prospective clinical trials and support clinicians in making more individualized dosing decisions. However, these advantages should be considered supportive only, as the present study evaluated PK/PD target attainment rather than clinical outcomes.

Several limitations of this study should be acknowledged. First, this is a simulation-based analysis using published PopPK parameters, not a prospective patient-level investigation. Real-world clinical outcomes are influenced by multiple complex factors beyond PK/PD exposure, and simulation findings cannot replace prospective clinical validation. Importantly, this study was explicitly designed as a model-based simulation analysis, rather than a clinical trial or a formal practice guideline. Its primary objective was to identify pharmacodynamically rational and clinically simplified dosing regimens using validated PopPK models, thereby providing a PK/PD evidence base to inform subsequent clinical validation efforts. All conclusions are hypothesis-generating and are not intended as direct clinical recommendations; prospective validation remains essential before any routine implementation. Second, the results may not be directly generalizable to patient populations with altered volume of distribution or clearance, such as obese, underweight, or critically ill individuals. Future studies should incorporate weight-stratified simulations (e.g., 50, 70, 100, and 120 kg) to enhance generalizability. Moreover, the current simulation framework did not account for comorbidities, microbiological heterogeneity, safety profiles, or actual clinical response.

## 4. Materials and Methods

### 4.1. Reconstruction of the PopPK Models

This was a simulation-based study derived from published aztreonam and avibactam PopPK models [11]. Model structure, covariate relationships, and parameter estimates were obtained from the published literature and implemented in Phoenix WinNonlin (Version 8.0, Certara USA, Inc., Radnor, PA, USA). Both compounds were described using two-compartment models after intravenous infusion. Creatinine clearance (CrCL) was the main covariate on clearance, and body weight was incorporated into clearance and distribution terms. The reconstructed PopPK models excluded the effect of infection type on clearance, and covariance between aztreonam and avibactam parameters was not incorporated. The parameters used are presented in Table S1.

### 4.2. External Validation of the PopPK Models

External validation of the reconstructed PopPK models for aztreonam and avibactam was conducted to assess predictive performance. The PopPK models were fully re-established using published parameters, including the two-compartment structural model, covariate relationships (creatinine clearance and body weight, population typical values, interindividual variability, and residual error models.

For external validation, data from two independent published clinical studies [12,13] were used. Subject characteristics (e.g., age, WT, CrCL) and dosing regimens (infusion duration, dose, interval) reported in those studies were input into the reconstructed models. Model-predicted plasma concentrations at corresponding sampling time points were generated by simulation and compared with observed concentrations from the literature.

Predictive performance was evaluated using standard metrics:

MPE% (mean prediction error percentage): Measures systematic bias; MAPE% (mean absolute prediction error percentage): Measures overall prediction accuracy [16]; F20/F30: Proportions of predicted concentrations within 20%/30% of observed values.

Acceptable predictive performance was defined as MPE% within ±20%, MAPE% ≤ 30%, F_20_ > 35%, and F_30_ > 50% [17,18].

### 4.3. Monte Carlo Simulation

Monte Carlo simulation was used to evaluate dosing performance across renal function strata. Virtual adult patients were simulated with a fixed body weight of 70 kg, which represents the WHO standard reference weight for adult pharmacokinetic simulations. These subjects were further divided into five CrCL subgroups: 101–120, 81–100, 51–80, 31–50, and 15–30 mL/min. For each subgroup, 1000 virtual patients were simulated. Interindividual variability for CL, V, Vp, and Q was sampled from a log-normal distribution as in the original PopPK model. Residual error: proportional + additive model as implemented in the reconstructed two-compartment model. Population typical values and interindividual variability terms were sampled repeatedly to generate concentration-time profiles and derived PK/PD metrics. Data processing and visualization were performed in R (Version 4.4.3, R Core Team, Vienna, Austria).

The evaluated scenarios included infusion durations of 1 h, 2 h, or 3 h; dosing intervals of q6h or q8h; a simplified maintenance regimen of 0.75/0.25 g q8h for patients with CrCL 15–30 mL/min versus the labeled regimen of 0.675/0.225 g q8h; and loading strategies of 2.0/0.67 g, 1.5/0.5 g, or no loading dose.

### 4.4. PK/PD Target Definition

Aztreonam and avibactam exhibit linear pharmacokinetic characteristics. The prespecified PK/PD targets were 60% fT > MIC for aztreonam (MIC = 8 mg/L) and 50% fT > CT for avibactam (CT = 2.5 mg/L), both of which represent clinically validated thresholds for aztreonam/avibactam against Enterobacterales, including MBL-producing carbapenem-resistant isolates [19,20].

For aztreonam, 60% fT > MIC is the well-established PK/PD target for β-lactams in severe bacterial infections, supported by phase 3 clinical data and regulatory guidelines. The MIC value of 8 mg/L corresponds to the EUCAST/CLSI clinical breakpoint for susceptible Enterobacterales [14,15,21].

For avibactam, 50% fT > 2.5 mg/L is the validated threshold for sustained β-lactamase inhibition. This target has been widely adopted in prior PK/PD investigations [14,15] and registration dosing trials.

A probability of target attainment (PTA) ≥ 90% was defined as the criterion for acceptable regimen performance, consistent with previously established standards [14,15,22,23]. Since aztreonam and avibactam are co-formulated and co-administered, their pharmacokinetic profiles were treated as perfectly correlated in all simulations. Accordingly, joint target attainment was defined as the primary pharmacodynamic endpoint for evaluating combination regimens. For any regimen to be considered acceptable, the predefined PK/PD targets for both aztreonam and avibactam must be met simultaneously. This approach ensures that the proposed simplified dosing strategies are valid for the fixed-dose combination as a whole.

Because the study relied exclusively on published model parameters and virtual patient simulations, without involving human participants or animals, ethics committee approval was not required.

## 5. Conclusions

In this PopPK-based Monte Carlo simulation study, selected aztreonam/avibactam dosing regimens could be simplified according to renal function without materially compromising PK/PD target attainment. A 2 h infusion may be considered a reasonable alternative for patients with CrCL 31–80 mL/min, and the 0.75/0.25 g q8h/2 h half-vial regimen could represent a potentially practical option for patients with CrCL 15–30 mL/min, though further evaluation is warranted. Loading-dose omission may be considered relatively reasonable in the CrCL 51–80 mL/min subgroup, whereas a more cautious approach is advisable in the CrCL 31–50 mL/min subgroup; in particular, omission of the loading dose is not recommended when a 2 h infusion is administered. Overall, these findings provide preliminary support for renal function-stratified administration strategies that may improve clinical practicality, but they still require prospective validation in real-world patients.

Future prospective clinical studies are warranted to validate these simulation-derived findings. Specifically, real-world investigations should evaluate the safety and efficacy of the renal function-stratified simplified regimens, including the 2 h infusion strategy and half-vial maintenance dose, in patients with varying degrees of renal impairment. Measuring plasma drug concentrations will help confirm PK/PD target attainment under these optimized regimens. Additionally, assessing clinical outcomes, microbiological eradication rates, and adverse events will further establish their clinical value. Inclusion of critically ill patients in the intensive care unit, as well as obese and renally impaired populations, will enhance the generalizability of the findings and support the translation of these simplified strategies into routine clinical practice.

## Figures and Tables

**Table 1:** Population pharmacokinetic model structure for aztreonam and avibactam.

Drug	CrCL > 80 mL/min	CrCL ≤ 80 mL/min
Aztreonam	V = tvV · (WT/70) · exp(ηV) Vp = tvVp · (WT/70) CL = tvCL · (WT/70)^0.75^ · [1 + (CrCL − 80) · Slope(CrCL on CL)] · exp(ηCL) Q = tvQ · (WT/70)^0.75^	V = tvV · (WT/70) · exp(ηV) Vp = tvVp · (WT/70) CL = tvCL · (WT/70)^0.75^ · (CrCL/80)^(CrCL on CL)^ · exp(ηCL) Q = tvQ · (WT/70)^0.75^
Avibactam	V = tvV · (WT/70) · exp(ηV) Vp = tvVp · (WT/70) · exp(ηVp) CL = tvCL · (WT/70)^0.75^ × [1 + (CrCL − 80) × Slope(CrCL on CL)] · exp(ηCL) Q = tvQ · (WT/70)^0.75^ · exp(ηQ)	V = tvV · (WT/70) · exp(ηV) Vp = tvVp · (WT/70) · exp(ηVp) CL = tvCL · (WT/70)^0.75^ · (CrCL/80)^(CrCL on CL)^ · exp(ηCL) Q = tvQ · (WT/70)^0.75^ · exp(ηQ)

WT, body weight (kg); V, central volume of distribution (L); Vp, peripheral volume of distribution (L); CL, clearance (L/h); Q, intercompartmental clearance (L/h); tv, population typical value; η, interindividual random effect.

**Table 2:** External Validation Results of the Reconstructed PopPK Models.

Reference	Drug	Dosing Regimen	Study Population (Weight, Renal Function)	Number of Data Points and Sampling Schedule	External Validation Results
#1 [12]	Aztreonam/avibactam	loading dose, 500/167 mg (30 min IV infusion) maintenance dose, 1500/500 mg (3 h IV infusion, q6h)	18 patients Weight (kg): 79.0 (55.0–100.0) CrCL (mL/min): 110.1 (40.2–182.4)	n = 6 (Aztreonam) n = 6 (Avibactam) Sampling time points: 1, 2, 3, 4, 5, and 6 h after multi-dose administration	MPE% = 16.8%; −18.8% MAPE% = 18.4%; 16.6% F_20_ = 50%; 66.7% F_30_ = 100%; 66.7%
#2 [13]	Aztreonam	1000 mg (100 mL), concomitantly infused over 30 min	13 healthy subjects Weight (kg): 70 CrCL (mL/min): 90	n = 11 (Aztreonam) Sampling time points: 0.4, 0.75, 1, 1.5, 2, 3, 4, 6, 8, 10, and 12 h after single-dose administration	MPE% = 13.9% MAPE% = 21.8% F_20_ = 63.6% F_30_ = 100%

Abbreviations: CLCR, creatinine clearance; IV, intravenous; q6h, every 6 h; MPE%, mean prediction error percentage; MAPE%, mean absolute prediction error percentage; F_20_, fraction of predicted concentrations within 20% of observed values; F_30_, fraction of predicted concentrations within 30% of observed values; CrCL, creatinine clearance; IV, intravenous. For aztreonam/avibactam, the validation metrics are presented in the order of aztreonam, avibactam.

**Table 3:** Probability of target attainment under alternative loading dose strategies.

Renal Subgroup	Analyte	Maintenance Regimen	Loading Strategy	Dose 1 PTA (%)	Dose 2 PTA (%)	Steady State PTA (%)	Acceptable?	Interpretation
CrCL 81–100 mL/min	Aztreonam	q6h, 3 h	2.0/0.67 g loading	98.02	96.66	97.08	Yes	PTA remained >90%.
	Avibactam			95.80	89.44	93.12		Second-dose PTA at the borderline
	Aztreonam	q6h, 3 h	No loading	96.86	97.08	97.08	Yes	PTA remained >90%.
	Avibactam			91.99	89.11	93.12		Second-dose PTA at the borderline
CrCL 51–80 mL/min	Aztreonam	q6h, 3 h	2.0/0.67 g loading	99.11	98.39	98.36	Yes	PTA remained >90%.
	Avibactam			99.80	98.29	99.44		
	Aztreonam	q6h, 3 h	No loading	98.30	98.36	98.36	Yes	Loading omission had little impact.
	Avibactam			99.30	98.21	99.44		
	Aztreonam	q6h, 2 h	2.0/0.67 g loading	95.77	94.80	94.76	Yes	Shortened infusion remained acceptable.
	Avibactam			95.09	91.67	93.41		
	Aztreonam	q6h, 2 h	No loading	94.60	94.76	94.76	Yes	Shortened infusion remained acceptable.
	Avibactam			91.71	91.13	93.41		Selective omission of loading supported
CrCL 31–50 mL/min	Aztreonam	q6h, 3 h	2.0/0.67 g loading	99.30	99.14	98.34	Yes	Labeled loading clearly acceptable.
	Avibactam			99.94	97.42	97.12		
	Aztreonam	q6h, 3 h	1.5/0.5 g loading	99.24	99.06	98.34	Yes	Packaging-matched loading acceptable.
	Avibactam			99.74	96.60	97.12		
	Aztreonam	q6h, 3 h	No loading	97.90	98.38	98.34	Yes	No loading acceptable under q6h/3 h.
	Avibactam			94.02	96.07	97.12		
	Aztreonam	q6h, 2 h	2.0/0.67 g loading	99.70	96.64	96.16	Yes	Shortened infusion acceptable
	Avibactam			98.26	91.92	91.44		
	Aztreonam	q6h, 2 h	1.5/0.5 g loading	99.28	96.52	96.16	Yes	Packaging-matched loading acceptable. Improved dose 1 exposure, but second-dose PTA at the borderline.
	Avibactam			96.46	89.94	91.44		
	Aztreonam	q6h, 2 h	No loading	95.14	96.10	96.16	No	The joint PTA of the first and second dose < 90%
	Avibactam			86.39	89.43	91.44		

CrCL, creatinine clearance; q6h, every 6 h. Aztreonam target = 60% fT > MIC; avibactam target = 50% fT > CT, where CT = 2.5 mg/L. PTA values < 90% were considered not acceptable under the prespecified criterion. Dose 1 PTA and Dose 2 PTA refer to the PTA values during the first and second dosing intervals after the initiation of treatment, respectively.

## Data Availability

The original contributions presented in this study are included in the article/Supplementary Materials. Further inquiries can be directed to the corresponding author.

## Supplementary Materials

The following supporting information can be downloaded at: https://www.mdpi.com/article/10.3390/antibiotics15060576/s1, Table S1: Parameters applied in the aztreonam/avibactam PopPK models.

## Abbreviations

The following abbreviations are used in this manuscript:

Abbreviation	Full term
ATM	aztreonam
AVI	avibactam
AUC	area under the concentration-time curve
CL	clearance
CrCL	creatinine clearance
CRE	carbapenem-resistant Enterobacterales
CT	pharmacodynamic threshold concentration
F_20_/F_30_	Proportions of predicted concentrations within 20%/30% of observed values.
FDA	U.S. Food and Drug Administration
fT > MIC	percentage of dosing interval that free drug concentration remains above the minimum inhibitory concentration
fT > CT	percentage of dosing interval that free drug concentration remains above the critical threshold concentration
MBL	metallo-β-lactamase
MBL-CRE	metallo-β-lactamase-producing carbapenem-resistant Enterobacterales
MIC	minimum inhibitory concentration
MCS	Monte Carlo simulation
MAPE%	mean absolute prediction error percentage
MPE%	mean prediction error percentage
PK/PD	pharmacokinetic/pharmacodynamic
PopPK	population pharmacokinetic
PTA	probability of target attainment
Q	intercompartmental clearance
V	central volume of distribution
Vp	peripheral volume of distribution
WT	body weight
