# Supplementary material for: Dosing Regimen Optimization of Aztreonam/Avibactam According to Renal Function Stratification: A Population Pharmacokinetic-Guided Simulation Study
Source: Antibiotics (Basel). 2026 Jun 5;15(6):576. doi: 10.3390/antibiotics15060576 (PMC13296099; doi:10.3390/antibiotics15060576)
Supplement: Supplementary file 1 [file antibiotics-15-00576-s001.zip › antibiotics-4289986-supplementary.pdf]

Supplementary Table S1. Parameters applied in the aztreonam/avibactam PopPK models [11].

ATM parameter	Estimate	AVI parameter	Estimate
tvCL_ATM [L/h]	5.00	tvCL_AVI [L/h]	10.3
tvV_ATM [L]	7.01	tvV_AVI [L]	12.6
tvQ_ATM [L/h]	9.41	tvQ_AVI [L/h]	4.82
tvVp_ATM [L]	6.12	tvVp_AVI [L]	6.95
CrCL on CL_ATM	0.502	CrCL on CL_AVI	1.06
Slope CrCL on CL_ATM	0.00383	Slope CrCL on CL_AVI	0.00313
η CL_ATM	0.4	η CL_AVI	0.4
η V_ATM	0.4	η V_AVI	0.6
-	-	η Q_AVI	0.31
-	-	η Vp_AVI	0.17
Additive RSV_ATM (mg/L)	0.197	Additive RSV_AVI (mg/L)	0.00621
Prop RSV_ATM (%)	12.5	Prop RSV_AVI (%)	20

Abbreviations: ATM, aztreonam; AVI, avibactam; CL, clearance; Q, intercompartmental clearance; V, central volume of distribution; Vp, peripheral volume of distribution; CrCL on CL, covariate parameter of creatinine clearance on clearance; η, interindividual variability; Prop, proportional; RSV, residual variability.
